# Screening and selection of camptothecin producing endophytes from *Nothapodytes nimmoniana*

**DOI:** 10.1038/s41598-021-90778-3

**Published:** 2021-05-27

**Authors:** I. A. H. Khwajah Mohinudeen, Saumya Pandey, Hemalatha Kanniyappan, Vignesh Muthuvijayan, Smita Srivastava

**Affiliations:** Department of Biotechnology, Bhupat and Jyoti Mehta School of Biosciences, Indian Institute of Technology Madras, Chennai, 600 036 India

**Keywords:** Biotechnology, Microbiology

## Abstract

Endophytic fungi with the ability to produce plant based secondary metabolites are a potential alternative for producing the host plant metabolite and to prevent natural plants from extinction. To isolate a high metabolite yielding endophytic strain from plants, hundreds of endophytic strains are screened and tested for product yield separately under axenic state, before shortlisting the potential endophyte, which involves huge time consumption. In this study, strategies for screening and selection of high camptothecin yielding endophytes from their natural habitat were proposed. A correlation was built between the camptothecin yield in the explants and the endophytes isolated from them. In addition, camptothecin yield was compared between the endophytes isolated from young and matured plants. Further, camptothecin producers and non-producers strains were compared for their tolerance toward camptothecin. The study indicates that high camptothecin yielding endophytes were isolated from high yielding explants and younger plants and they were more tolerant to camptothecin in comparison to non-camptothecin yielding endophytes. Thus, choosing a young and high yielding explant for endophyte isolation, and use of camptothecin as a selective agent in the growth medium, can be instrumental in screening and selection of high camptothecin yielding endophytes from nature in relatively less time.

## Introduction

Plants contribute to more than 25% of the approved drugs in the worldwide drug market and to 11% of WHO acknowledged generic drugs^1^, making them an indispensable source for medicines. As a result of this 500,000 tons of plant material is being traded worldwide annually resulting in only 1.4% of them remaining on the earth’s surface^2^. Camptothecin (CPT) is one such plant derived metabolite which is in high demand across the world for its anti-cancer activity. CPT is a monoterpene pentacyclic cytotoxic quinoline alkaloid, which binds with the DNA topoisomerase I complex and forms a physical barrier which prevents DNA replication and eventually resulting in cell death^3,4^. CPT is majorly extracted from two plants, *Camptotheca acuminata* and *Nothapodytes nimmoniana*, for commercial applications^5^. Both these plants are critically endangered due to over-harvesting in order to meet the ever increasing market demand of CPT^6^. Therefore, a need for an alternative source always prevails to prevent such medicinal plants from extinction. Endophytes, the microorganisms that reside within the plants, have attained the ability to produce their host plant based metabolite. Since the first report of endophyte producing host plant associated metabolite^7^, they have been explored as a potential alternative to natural plant biomass owing to the feasibility of sustainable bioprocess development and economical scale-up of microbial fermentations due to their fast growth rates. However, selection of an endophyte with high yield and significant activity from the plethora of endophytic population isolated from the plant, is cumbersome and time-consuming. An endophyte *Entrophospora infrequens* producing CPT was successfully isolated from *N. nimmoniana* after screening 52 strains of endophytic fungi^8^. Similarly, *Fusarium solani* S-019, a potent strain that displayed impressive cytotoxic activity was obtained after screening 94 endophytic fungi isolated from *C. acuminata*^9^. In our previous study as well, an endophyte *Alternaria burnsii*^10^, demonstrated sustainable production of CPT even after 12 subculture cycles in suspension culture, was obtained after the screening of 132 endophytic strains isolated from the same plant of *N. nimmoniana.* All these cultures were grown separately in suspension and CPT extraction was done from their biomass to estimate the yield of CPT from each strain for comparison and selection of a high yielding strain. This study took more than a year to screen the cultures individually for CPT production, in their axenic state. Hence, we felt a need for developing less time consuming screening methodology to select high CPT yielding endophytes from nature. Thus our objectives were: (1) to determine whether the CPT content in the natural plant parts is correlated to the endophytes isolated from them, to demonstrate that high yielding endophytes could be originating from high yielding explants. (2) To test the hypothesis that the high yielding endophytes could be more tolerant towards CPT than the non-CPT yielding endophytes. (3) To determine the cytotoxic potential of the extracts from CPT yielding strains was investigated and compare them against the non-CPT yielding strains.

## Results

### Isolation, identification and molecular characterization of endophytes

The endophytic fungi isolated from the *N. nimmoniana* plant parts were grouped based on the plant and the type of explant from which they were isolated. From the 132 isolates, we obtained certain high CPT yielding endophytes reported in our previous study^10^. In this study the endophytes which produced high CPT yield were compared with those endophytes which were isolated from the same explant and did not produce detectable level of CPT (Supplementary Table S1). The high yielding endophytes were characterized in our previous study and their complementary pairs (the non-CPT yielding endophytes isolated from the same explant) chosen for this study have been characterized here. The highest CPT yielding strain (CPT1) was isolated from the petiole region of the plant and another strain isolated from the same petiole which could not produce CPT was considered as its complementary pair (NCPT1) for this study. Similarly, the second and third highest yielding strains (CPT2 & CPT3) were isolated from the leaf explants, while the corresponding non-CPT yielding strains from the same explants were taken as their non-CPT producing counterpart (NCPT2 & NCPT3), respectively. Genomic DNA isolated from each of them was amplified using ITS1 and ITS4 primers for molecular identification. The amplified products were purified and found to be 500–700 bp in agarose gel electrophoresis (Supplementary Fig. S1), which corresponds to the ITS region of the fungi. The amplified template was sent for sequencing and a consensus sequence was generated by aligning the forward and reverse sequence obtained. This consensus sequence containing ITS1, 5.8S and ITS2 regions were compared with the DNA sequence database using the nucleotide BLAST algorithm provided by (National Center for Biotechnology Information) NCBI. As shown in Table 1, the search results that provided highest score and coverage were used to identify the respective endophyte’s genus and species (Table 1). The strain NCPT1 which was isolated from the petiole region was found to be *Bipolaris subramanianii* and the strains NCPT2 and NCPT3 isolated from the leaf regions were found to be *Diaporthe melonis* and *Colletotrichum cobbittiense,* respectively.

**Table 1:** Endophytic strains yielding CPT and their non-CPT yielding pair isolated from the same explant.

Endophytic strain	Explant	Camptothecin yield (µg/g)	Species	Genbank accession number
CPT1	Petiole	426.7 ± 33.6	*Alternaria alstroemeriae*	MN795638
CPT2	Leaf	403.3 ± 41.6	*Alternaria burnsii*	MN795639
CPT3	Leaf	269.4 ± 53.9	*Alternaria alstroemeriae*	MN795640
NCPT1	Petiole	ND	*Bipolaris subramanianii*	MW450846
NCPT2	Leaf	ND	*Diaporthe melonis*	MW450847
NCPT3	Leaf	ND	*Colletotrichum cobbittiense*	MW450848

^*#*^*ND* not detectable.

### A high yielding endophyte from a high yielding explant

**Figure 1:**
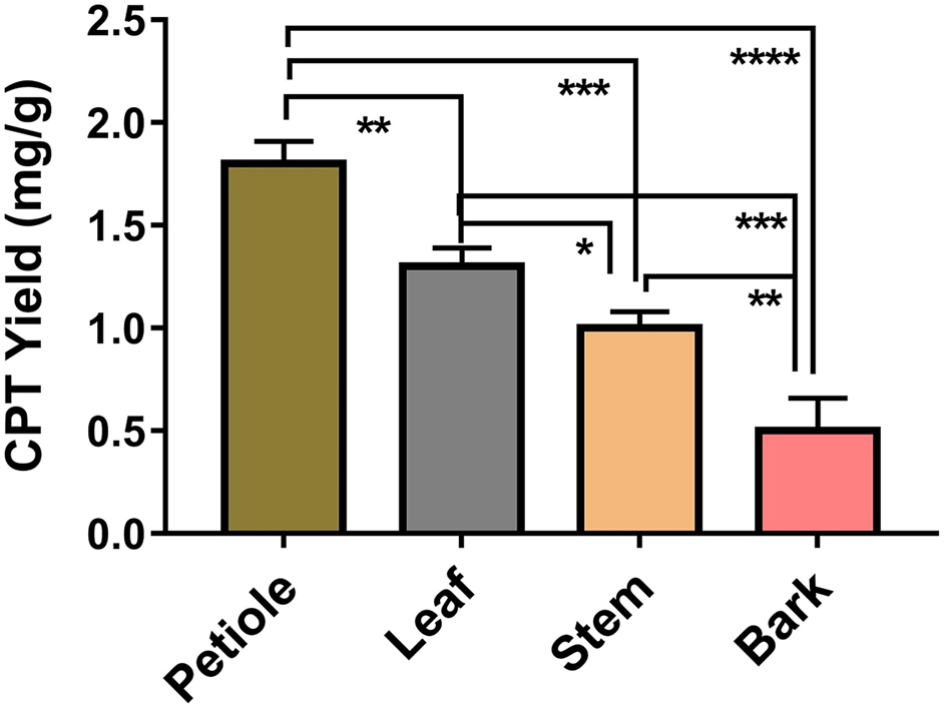
Camptothecin yield obtained from the different explants (plant parts) of *N. nimmoniana* plant used for the isolation of CPT yielding endophytes.

A study was done to analyze the distribution of CPT yield across the plant parts (leaves, petiole, stem and bark) of *N. nimmoniana* used as explants for the isolation of endophytes. This analysis was done during the monsoon season at the same time at which endophytes were isolated. Petiole showed the highest yield of 1.75 ± 0.1 mg/g, followed by leaves with a yield of 1.32 ± 0.09 mg/g (Fig. 1). The average yield in stem was 1.01 ± 0.08 mg/g and in the bark was 0.53 ± 0.22 mg/g. The data were found to be statistically significant with a p-value < 0.05. The CPT yields reported in literature from the parts of *N. nimmoniana* natural plant varied from 0.1 to 10 mg/g^11–16^. It is to be noted that the highest yielding endophyte was isolated from the petiole explant which demonstrated highest CPT yield (426.7 ± 33.6 µg/g) during the period of isolation followed by two leaf derived endophytes with CPT yields of 403.3 ± 41.6 and 269.4 ± 53.9 µg/g respectively. This could be due to the increased tolerance of CPT in high yielding endophytes, simultaneously leading to high yields in potential endophytes (CPT- yielding).

**Figure 2:**
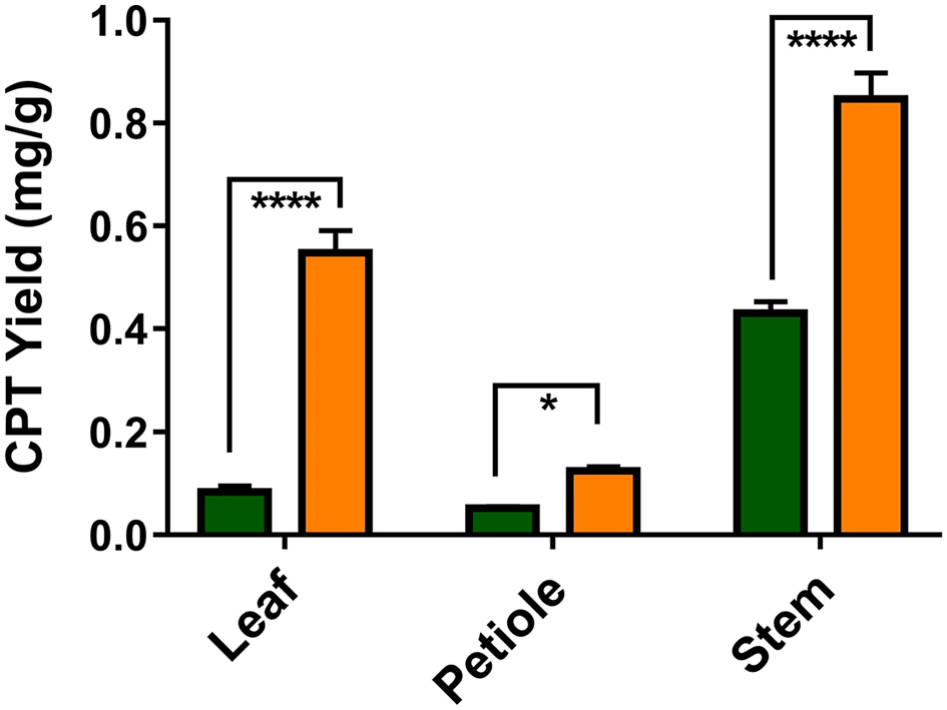
A comparison of CPT yield in different explants (plant parts) obtained from young plantlets and matured plants of *N. nimmoniana* ( Mature Plant  Young Plant).

Interestingly, all the high CPT yielding endophytes were isolated from young plantlets which were saplings of ~ 1ft height. So a separate study was done in a different season (summer) to check if there is correlation in CPT yield between the parts of young plantlets and matured (tress up to 7ft height) *N. nimmoniana* plant. It was observed that all the explants (leaf, petiole and stem) showed significantly higher yield in younger plants (seedlings) than the matured plants (Fig. 2). The younger plant parts had at least two fold higher yield when compared with the matured plant parts (Fig. 2). The data was found to be significant with a p-value of < 0.05 when a two-way ANOVA was performed.

### High yielding endophytes are more tolerant toward camptothecin toxicity than non-CPT yielding endophytes

**Figure 3:**
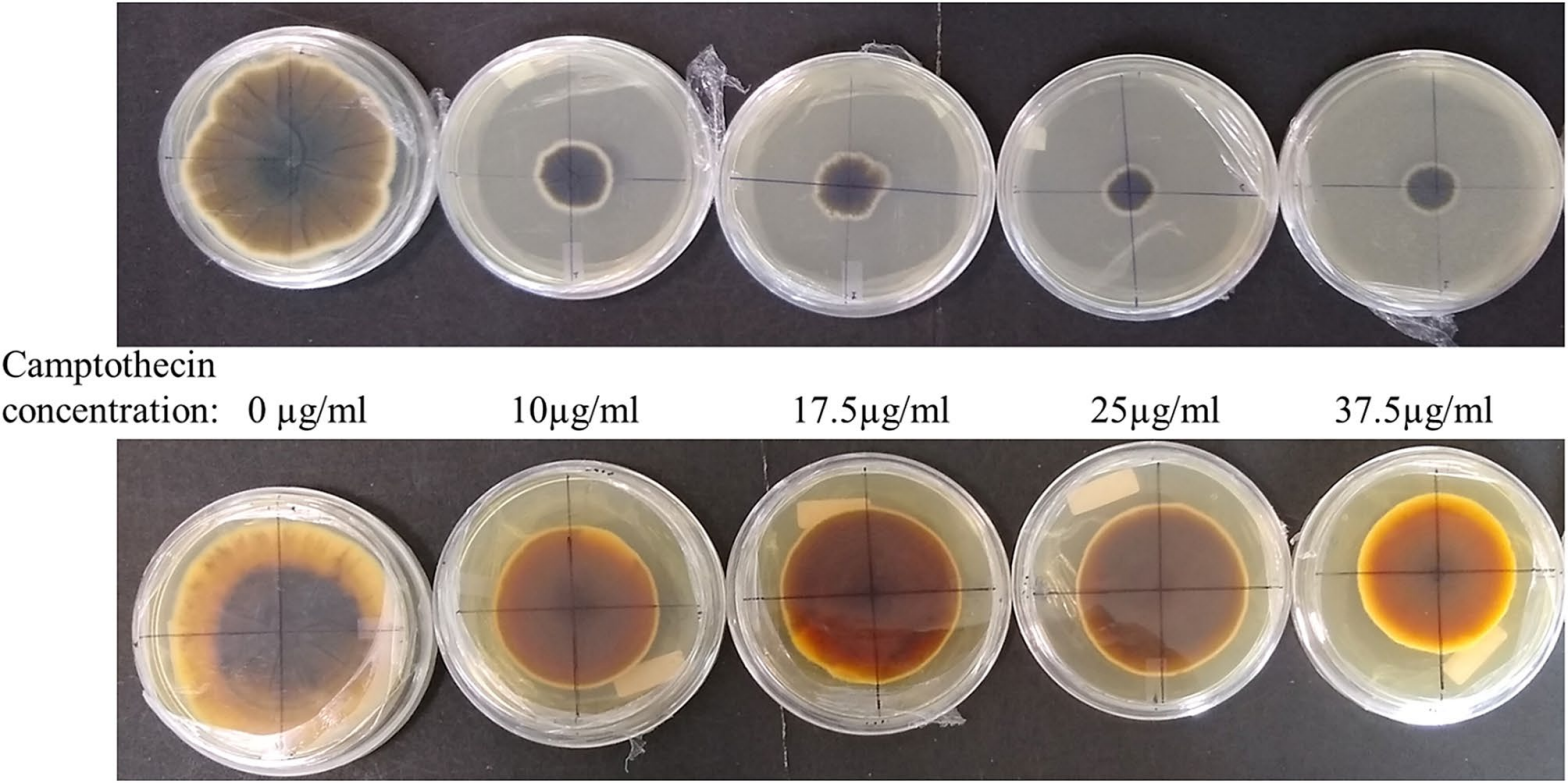
Comparison of camptothecin tolerance in non camptothecin producing strain (NCPT1) (top) with camptothecin producing strain (CPT1) (bottom) in increasing concentrations of camptothecin.

**Figure 4:**
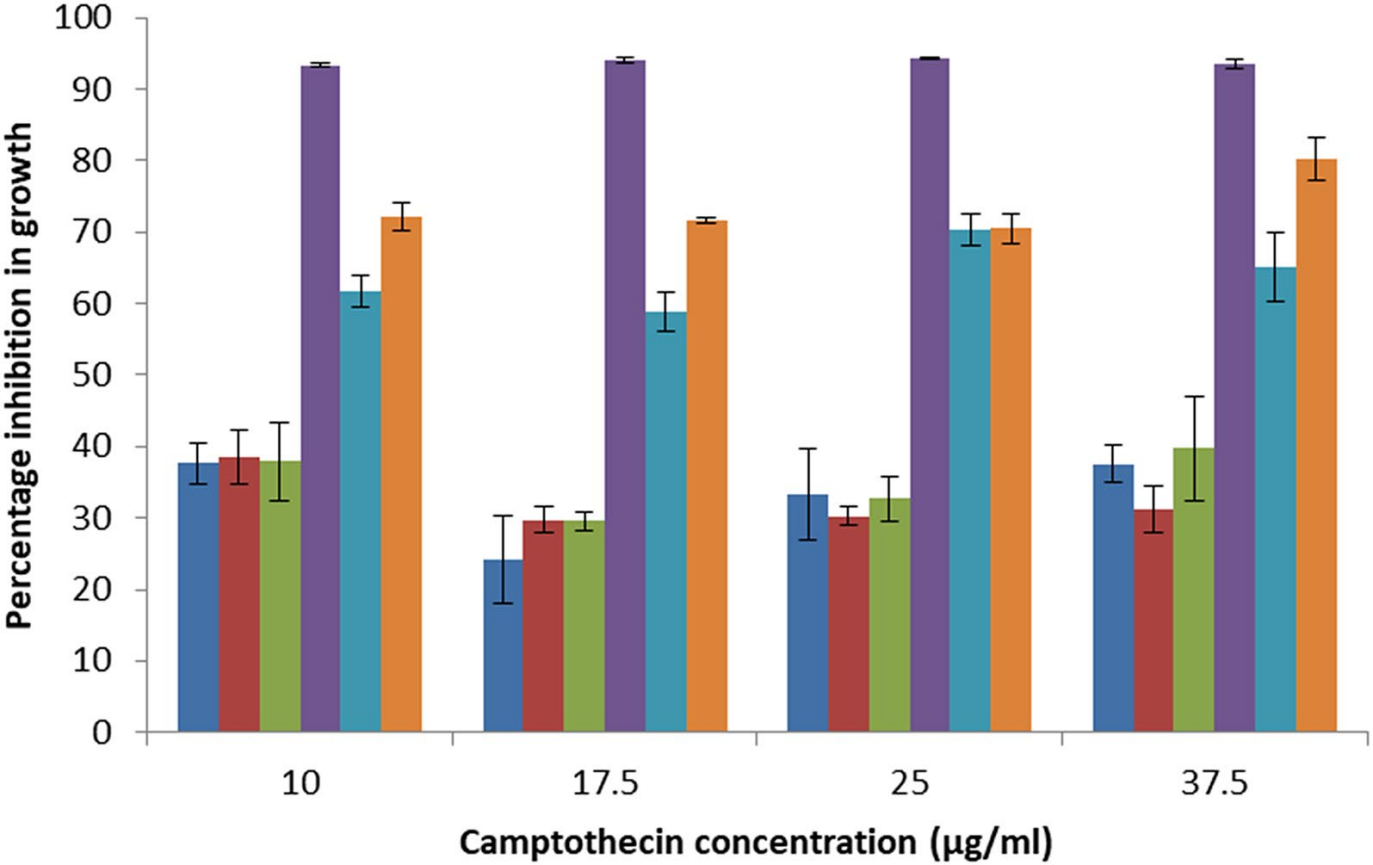
Percentage inhibition in growth of non camptothecin producing strains ( NCPT1,  NCPT2 and  NCPT3) in comparison to the camptothecin producing strains ( CPT1,  CPT2 and  CPT3) with increasing concentrations of standard camptothecin in media.

The CPT yielding endophytic strains (CPT1, CPT2 and CPT3) were compared against the non-CPT yielding endophytic strains (NCPT1, NCPT2 and NCPT3) for their tolerance towards varying concentrations of standard CPT. Growth profile of all the endophytic strains showed inhibition at varying range in the presence of standard CPT when compared with control (Supplementary Fig. S2). It was observed that at all the initial concentrations of CPT tested, greater inhibition in growth was observed in all the non-CPT yielding strains from the control than that in the CPT yielding endophytes (Fig. 3). A plot between the percentage reduction in growth of the strain with the increasing concentrations of CPT shows that, at all the concentrations of CPT tested, nearly twofold more reduction in growth was observed in all the non-CPT yielding strains than that in the CPT yielding counterparts isolated from the same explants (Fig. 4).

### Cytotoxicity analysis of endophytes isolated from *N. nimmoniana*

**Figure 5:**
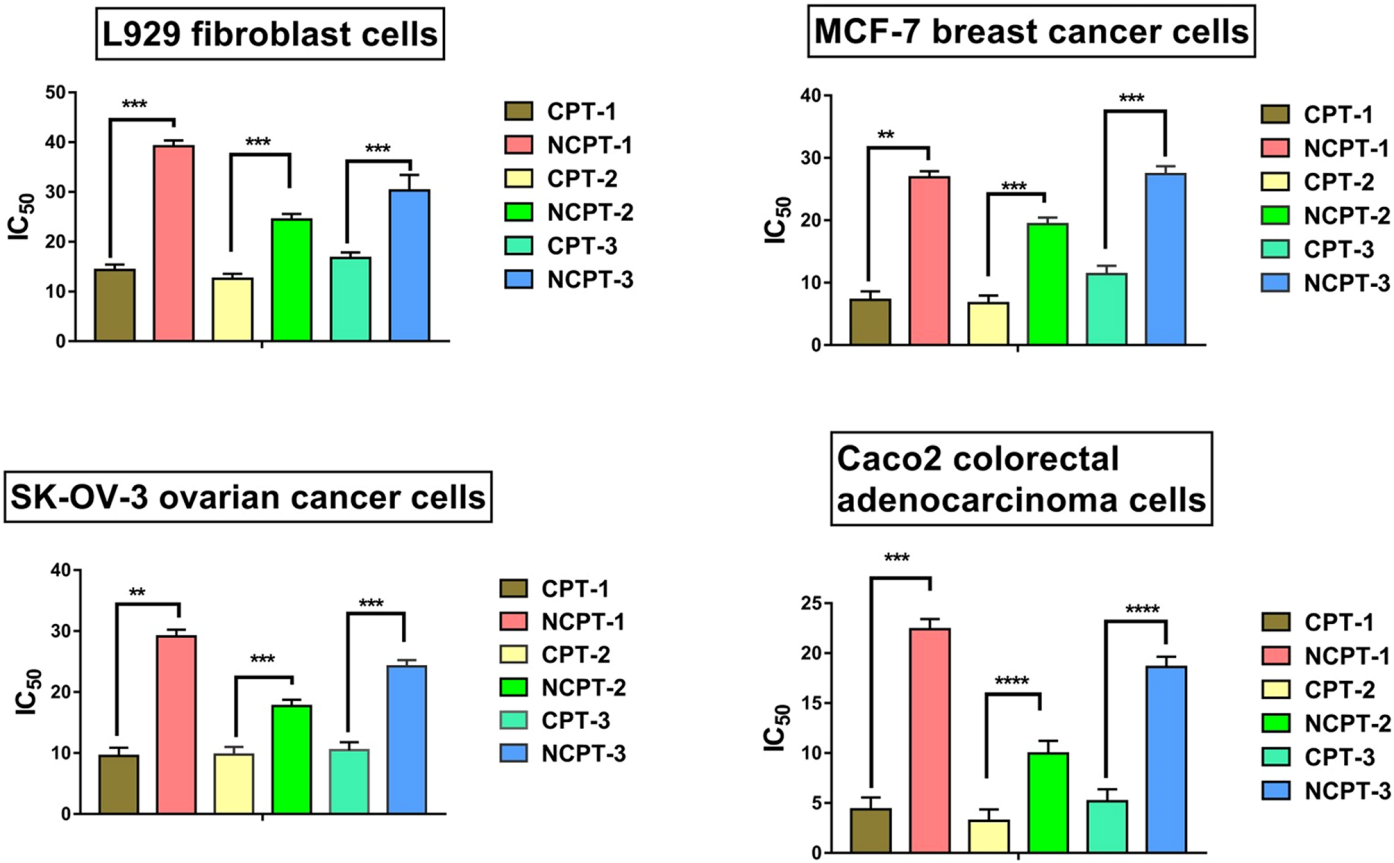
Comparison of cytotoxicity between camptothecin producing strains ( CPT1,  CPT2 and  CPT3) and non-camptothecin producing strains ( NCPT1,  NCPT2 and  NCPT3) on cancerous (MCF-7, SK-OV-3, Caco-2) and non-cancerous (L929) cell lines.

The CPT producing endophytic extract was compared with the extract from their non-CPT producing counterpart isolated from the same explant, to check if presence of CPT in the extract has a significant effect on their cytotoxic potential. Four cell lines were chosen for this study, which includes a non-cancerous L929 fibroblasts cells and three cancerous cell lines MCF-7, SK-OV-3 & Caco-2. It was found that in all cases the extract from CPT producing strains was found to be significantly more cytotoxic against the cancerous and non-cancerous cell lines tested when compared with their respective non CPT producing pairs (Fig. 5). The extract of CPT producing endophytic strain, CPT1 (*Alternaria alstroemeriae*), isolated from the petiole region of *N. nimmoniana* showed an IC_50_ value of 14.37, 7.25, 9.51 and 4.36 µg/ml against L929, MCF-7, SK-OV-3 & Caco-2 cell lines respectively. On the other hand the extract from the non-CPT producing endophytic strain NCPT1 (*Bipolaris subramanianii*), isolated from the petiole region of the same plant had an IC_50_ value of 39.2, 26.88, 29.13 and 22.41 µg/ml towards L929, MCF-7, SK-OV-3 & Caco-2 cell lines respectively. Similarly, in case of the other pair of strains isolated from the leaf regions of the plant, the extract from the CPT producing strains CPT2 (*Alternaria burnsii*) & CPT3 (*Alternaria alstroemeriae*), were found to be twice more cytotoxic than their respective non CPT producing counterparts, NCPT2 (*Diaporthe melonis*) & NCPT3 (*Colletotrichum cobbittiense*). This increased cytotoxicity towards the cancerous and non-cancerous cell lines should be contributed by the presence of CPT in the CPT producing strains.

## Discussion

Endophytes are looked forward as one of the potential alternative source for plant based metabolite production and this is evident from the increasing research in the field of endophytes in the recent years. However, screening a large number of endophytes from plants in order to obtain a high yielding strain is an arduous and time consuming task. In our previous study, we isolated 132 endophytic strains from the plant parts of *N. nimmoniana* and screened them for CPT production. This process of screening happened more than a year to grow the strains under in vitro condition and extract camptothecin from them for quantification. Therefore, we realize the need for rapid screening technique(s) that could help in reducing the time taken for bioprospecting of potential endophytes.

Host plant stimulus is reported to play a key role in the production of metabolite by the endophytes^17^. Therefore, the relative level of camptothecin in the plant parts that were used as explants for endophyte isolation was estimated. At the same time of isolation of endophytes (monsoon season), plant parts were also analyzed separately for estimation of CPT level in them. It was found that higher CPT yielding endophytes were isolated from the plant parts containing high concentration of CPT in them. Another interesting fact was that all the highest yielding strains were obtained from the parts of young plantlets. Hence, to compare the level of camptothecin between matured plants and young plantlets, a separate study was performed in a different season (summer) to compare the level of camptothecin between parts of young and matured plants. Also, seasonal variation in the CPT yield of the plant parts could be observed, where higher CPT yield was obtained in the monsoon season than in the summer. It is to be noted that seasonal variation in the yield of secondary metabolites in plants is well known and endophyte distribution is also reported to be subjected to seasonal variation in a plant^18,19^. Considering this fact, the objective of this study was only to compare the CPT yield between young and matured plants and not across the types of tissues. The comparative analysis demonstrated that younger plant parts have much high levels of camptothecin in comparison to matured plant parts. This is also in line with earlier reports where the CPT yield in *C. acuminata* plant was also found to vary with age^20^ and younger leaves of *C. acuminata* were reported to have much higher yields of CPT than older leaves^21^. The results indicate that CPT yield variation among the potential endophytes isolated from a plant follows the same trend as the CPT yield variation among the different tissue types of that plant. Therefore, screening of high CPT yielding plants (and explants) can result in bioprospecting of high CPT yielding endophytes. This is similar to the fact that a high yielding plant when used as a source for multiplying plantations and for developing cell lines or in vitro production systems, can lead to the development of a high yielding plantlet/cell line/in vitro system^13^. As a future scope in the study, analysis of camptothecin biosynthesis pathway in these CPT yielding endophytes can further support these screening methodologies for selecting potential endophytes.

As camptothecin is known to have potential antifungal activity^22,23^, it was hypothesized that the fungus producing the antifungal compound could have higher tolerance towards the compound in comparison to other non-producing fungi. Hence, to prove the hypothesis, we chose the three highest CPT yielding strains from our previous study and compared their tolerance toward CPT with their respective non CPT producing strain isolated from the same plant and explant. The CPT yielding endophytic strains were compared against the non-CPT yielding endophytic strains for their tolerance toward the growth inhibitory effect of CPT. As expected, the chosen CPT producing strains were more tolerant towards CPT than the non-CPT producing strains. This result was in line with our hypothesis that endophytes with higher yield of CPT are more tolerant towards the toxic metabolite. It is to be noted from earlier reports that some endophytes isolated from a taxol producing plant were found to be tolerant toward taxol^24,25^. A modification in the aminoacid sequence of tubulin was observed in these endophytes indicating a possible reason for increased tolerance towards taxol^24^. While in case of CPT it is reported that endophytes producing CPT were inhibited by exogenous CPT^26^. However, in our study, the rate of inhibition was higher in case of non-CPT producing strains (60–90% inhibition) in comparison to the CPT producing strains (25–40% inhibition). Although expensive but this feature can be used for rapid screening of high CPT yielding endophytes from plants. Nonetheless, the fact that every plant is a repository of endophytes, the technique can save the time and money spent in long-duration experiments required for the qualitative and quantitative analysis of CPT from their axenic cultures.

Endophytic extracts are known to have various bioactivities including antimicrobial, anti-malarial, antioxidant, antiviral and anticancer activities^27–29^. Hence, the endophytic extracts from the strains isolated in this study are also expected to be cytotoxic against the cancerous and non-cancerous cell lines. However, the level of cytotoxicity is believed to be much higher if in case the endophyte has the potential to produce CPT, since CPT is one of the major anticancer compounds used worldwide as a precursor for manufacturing anticancer drugs and is known for its high level of cytotoxicity^30,31^. Hence, the cytotoxic effect of the CPT producing endophytic extract was compared with the extract from their non-CPT producing counterpart isolated from the same part and plant. As anticipated, the CPT producing strains demonstrated more cytotoxicity toward both the cancerous and non-cancerous cell lines chosen in the study. Although, there are reports on other Alternaria species’ extracts showing cytotoxic activity on cancerous cell lines, but none of these reports indicate camptothecin production by these cultures. Moreover, the results obtained in this study indicate that the order in which these endophytes could be ranked on the basis of their CPT yield was the same in which their extracts could be ranked for the cytotoxic potential on cancer cell lines. At the same time, irrespective of the possibility of other compounds present in the culture extracts tested, all the non-CPT producing endophytes in the study demonstrated significantly low cytotoxicity (IC_50_ values) in comparison to that in the CPT producers. This suggested that the content of CPT in the culture extracts had a significant impact on their cytotoxic potential against cancer cell lines.

Based on the results, we propose certain screening strategies which can help in rapid isolation of CPT yielding endophytes from natural plants. Since higher CPT yielding endophytes were obtained from higher CPT yielding explants, it can be beneficial (w.r.t time, effort and money) to screen the CPT yield in the explants before bioprospecting for high yielding endophytes. This study indicates that a high CPT yielding explant from a young plantlet (of *N. nimmoniana*) should be preferred for bioprospecting of high CPT yielding endophytes. Additionally, high CPT yielding strains of endophytes demonstrate high CPT tolerance. Hence, for screening of (high) CPT-yielding endophytes, CPT itself can be added in the growth medium as a selective agent at lethal concentrations to non-CPT yielding endophytes. Extract of higher CPT—tolerant endophytes could also demonstrate higher toxicity toward cancer cell lines than less tolerant endophytes, which could be supported by the estimation of higher CPT yields in more tolerant endophytes. This further strengthened our hypothesis.

## Methods

### Isolation, identification and molecular characterization of endophytes from *Nothapodytes nimmoniana*

In our previous study endophytes were isolated from leaves, petioles, bark and stem regions of six different *N. nimmoniana* plants from the campus of University of Agricultural Science, Bengaluru (13.0762°N, 77.5753°E)^10^. The explants were washed and surface sterilized with 1% (v/v) sodium hypochlorite followed by 70% (v/v) ethanol. Surface sterilized explants were incubated at 28 °C on plates made with solidified potato dextrose agar medium (HiMedia, Mumbai) for 7 days. Morphologically distinct endophytes were separated from the explants and grown on fresh plates. Suspensions were initiated for each strain in 250 ml Erlenmeyer flask containing 50 ml of potato dextrose broth medium. Each of the culture suspensions were incubated at 28 °C and 120 rpm for 8 days and the biomass was harvested separately and dried in hot air oven. CPT extraction was done from 0.3 g of dried biomass and then analyzed in HPLC to determine the CPT yield as mentioned in the analytical methods.

To characterize the endophytic strains, fresh mycelial mat (biomass) obtained from suspension culture was taken and washed with ethanol and ground to fine powder using a mortar and pestle with liquid nitrogen. DNA was isolated from the ground powder using DNA isolation Kit following the manufacturer’s protocol (HiMedia, Mumbai). The isolated DNA was amplified using PCR with ITS1 forward primer (5´ TCCGTAGGTGAACCTTGCGG 3´) and ITS4 reverse primer (5´ TCCTCCGCTTATTGATATGC 3´)^32^ using Taq DNA polymerase 2 × master mix red (Ampliqon, Denmark). PCR amplification was done in a Veriti thermocycler (Applied Biosystems, USA). PCR conditions used for amplification were as follows: initial denaturation at 95 °C for 5 min followed by 25 cycles of denaturation at 95 °C for 30 s, annealing at 57 °C for 40 s, extension at 72 °C for 30 s and a final extension at 72 °C for 5 min. The amplified PCR products were further purified with a Qiaquick PCR purification kit (Qiagen, California, USA) following the manufacture’s protocol. Agarose gel electrophoresis (0.8% w/w agarose) was performed by loading 10 µl of the PCR product along with 2 µl of gel loading dye into the wells. The system was allowed to run for 30 min at 50 V and then visualized under UV transilluminator (GelDoc, BioRad, Italy). The purified PCR products were sequenced using AB 3130 Genetic Analyzer (Applied Biosystems, Foster City, CA) by Sanger dideoxy method.

The forward and reverse sequences were aligned to obtain a consensus sequence containing the ITS1, 5.8S and ITS4 regions in them. These consensus sequences were submitted as queries in nucleotide BLAST algorithm from NCBI (National Center for Biotechnology Information) for comparison against the database of sequences. The search was restricted to only to the sequences from type material for increased confidence in identification^33^. A phylogenetic tree was constructed with MEGA7^34^ by using maximum likelihood method based on Jukes-Cantor model^35^ (Supplementary Figure S3) and the sequences were also deposited in NCBI.

### Sampling of *N. nimmoniana* plant for camptothecin analysis

In compliance with local and national regulations, different plant parts (explants), like leaves, petiole, stem and bark region, from which endophytes were isolated were collected from the horticulture of University of Agricultural Science, Bengaluru (13.0762°N, 77.5753°E) for estimation of CPT content. The plant specimen was authenticated as *N. nimmoniana* (J.Graham) Mabb. of the family Icacinaceae and deposited in Foundation for Revitalisation of Local Health Traditions (FRLH) herbarium, Bengaluru (FRLH 122,013) (Courtesy Dr. S. Noorunnisa Begum, Curator – FRLH Herbarium). These fresh plant parts were washed well with running tap water and then washed thoroughly with distilled water. These washed fresh plant parts were dried in hot air oven at 60 °C for 48 h. Extraction of CPT and quantification was performed in triplicate by means of HPLC as explained in the analytical methods section. Data was analyzed by means of One-way ANOVA followed by Tukey's post hoc test using GraphPadPrism version 6 for Windows, GraphPad Software, La Jolla California USA, www.graphpad.com.

As all the high yielding endophytes were found to be isolated from young plantlets which were saplings of ~ 1ft height, a separate study was done in a different season (summer) to compare CPT yield between the parts of young plantlets and matured (tress up to 7ft height).

*N. nimmoniana* plant. Leaf, petiole and stem regions were collected from both young and matured plants and their CPT yield was analyzed. Five biological replicates were taken for each type of explant from both young and matured plants, and three analytical repeats were done for each biological replicate for estimating the statistical significance of the data. Data was analyzed by a Two-way ANOVA followed by Tukey's post hoc test using GraphPad Prism version 6 for Windows, GraphPad Software, La Jolla California USA, www.graphpad.com.

### Evaluation of tolerance/resistance toward camptothecin toxicity in CPT yielding and non-CPT yielding strains

The CPT yielding endophytic strains (CPT1, CPT2 and CPT3) were compared against the non-CPT yielding endophytic strains (NCPT1, NCPT2 and NCPT3) to evaluate their tolerance toward the growth inhibitory effect of CPT. Fresh slants were initiated by streaking 10 μl of the glycerol stocks of the strains on test tube slants containing 5 ml of Potato dextrose agar medium. Slants were incubated at 28 °C for 7 days and then washed with saline (0.9% w/v NaCl) to obtain the fungal spores and mycelia from the slants. From these washed cells, 10 μl is inoculated into sterile petri plates (90 mm diameter) containing PDA medium. Fungal strains in petri plates were allowed to grow at 28 °C for 7 days. Using a sterile cork borer, agar disc (5 mm diameter) were made from the actively growing regions of the 7 day old culture and inoculated onto fresh plates with PDA medium containing CPT at different concentrations (10, 17.5, 25, 37.5 μg/ml). Plates containing only PDA medium without CPT were used as control. The culture plates were maintained at 28 °C and the radius of the mycelial growth (mm) was measured using digital Vernier caliper after every 12 h till the complete growth (full coverage of the plate with growing mycelia) was visualized on the control plates. The reduction in the radial growth of the mycelia in comparison to the control was calculated.

### In vitro anticancer activity of CPT yielding and non-CPT yielding endophytic strains

Cytotoxic effects of the CPT yielding and non-CPT yielding endophytic extracts were investigated using the alamarBlue assay using a previously described protocol^36,37^. The assay was performed on three cancerous cell lines [Caco-2 human colorectal adenocarcinoma cells, MCF-7 breast cancer cells and SK-OV-3 human ovarian cancer cells] and a non-cancerous cell line [L929 fibroblast cells] for up to 72 h. The cells were procured from the NCCS (National Centre for Cell Science), Pune, India and DMEM (Dulbecco's Modified Eagle's Medium) with 10% FBS (Fetal Bovine Serum) and 1% penicillin–streptomycin, obtained from Gibco, were used as the culture medium for all the cells. The cells were maintained in an incubator at 37 °C with 5% (v/v) CO_2_ atmosphere. Once the cells attained 80% confluency, the cells were counted and used for the cytotoxicity analysis. The cells were seeded at a density of 1 × 10^4^ cells per well in a 96-well culture plate and maintained in an incubator at 37 °C for 24 h at 5% (v/v) CO_2_ atmosphere. After the cell monolayer is achieved, the extracts at different concentrations (0.2, 0.4, 0.8, 1.6, 3.2, 6.4, 12.8, 25.6 and 51.2 μg/ml) diluted with serum free media. Cells without extract were treated as control.

The plates were incubated at 37 °C for 48 and 72 h in a fully humidified atmosphere of 5% (v/v) CO_2_. After the incubation, 100 μL of 5 mg/mL alamarBlue solution was added and the mixture was incubated for 4 h at 37 °C. This solution was then transferred to fresh 96-well plates and the cell viability was determined by measuring the optical density of the test (a culture medium containing cells and crude CPT extract), blank (culture medium), and vehicle control at wavelengths of 570 nm and 600 nm using a microplate reader.

The cell viability was calculated using the formula with the measured absorbance^38^.

$$CV = \left( {\frac{{(Abs_{600} E^{\prime} \times Abs_{570} Sample) - (Abs_{570} E \times Abs_{600} Sample)}}{{(Abs_{600} E^{\prime} \times Abs_{570} Positive\,control) - (Abs_{570} E \times Abs_{600} Positive\,control)}}} \right)$$

where *CV* = cell viability, *E *= molar extinction coefficient of oxidized alamarBlue at 570 nm, *E*′ = molar extinction coefficient of oxidized alamarBlue at 600 nm.

The values obtained for cell viability in the different crude CPT extract were normalized to that obtained in the control plate. The final value was represented as fold cell viability.

### Analytical methods

#### Extraction of camptothecin

The dried biomass (0.3 g) was dissolved in 20 ml of distilled water and homogenized using a mortar and pestle, followed by liquid – liquid extraction, repeated thrice using 50 ml of chloroform: methanol solvent mixture (4:1). The organic layer with dissolved camptothecin was then collected and removed using a rotary evaporator. The dried camptothecin extract was re-suspended in 1 ml of DMSO: methanol (1:50) and filtered through 0.2 µm filter for further analysis^10^.

#### Quantification of camptothecin by high performance liquid chromatography (HPLC)

Twenty microliter of the extract sample was injected in a reverse phase HPLC (LC-20AD, Shimadzu, Japan) at a flow rate of 0.8 ml/ min using 25% acetonitrile as the mobile phase. ODS Hypersil gold column (Thermo Scientific) with a particle size of 5 µm was used as the stationary phase at a temperature of 30 °C. Absorbance of camptothecin was measured at 254 nm by a photodiode array detector^10^. Concentration of camptothecin in the sample was estimated using a standard plot of area vs. known concentration of camptothecin, generated using authentic samples of camptothecin (> 95% purity, Sigma Aldrich, St. Louis, MO, USA ).

### Statistical analysis

All experiments were performed in triplicates. Statistical analysis was performed using v6.0, GraphPad Prism software, USA. The experimental data were analyzed with either one-way or two-way ANOVA, followed by a Tukey post hoc test. The values of *p < 0.05, **p < 0.01, ***p < 0.001 and ****p < 0.0001 were considered to be statistically significant.

## Supplementary Information

Supplementary Information.
