# Supplementary material for: Screening and selection of camptothecin producing endophytes from Nothapodytes nimmoniana
Source: Sci Rep. 2021 May 27;11:11205. doi: 10.1038/s41598-021-90778-3 (PMC8159990; doi:10.1038/s41598-021-90778-3)
Supplement: Supplementary file 1 — Supplementary Information. [file 41598_2021_90778_MOESM1_ESM.docx]

**Supplementary data**

**Screening and selection of camptothecin producing endophytes from *Nothapodytes* *nimmoninana***

I. A. H. Khwajah Mohinudeen^1^, Saumya Pandey^1^, Hemalatha Kanniyappan^1^, Vignesh Muthuvijayan^1^, Smita Srivastava^1^*

^1^Department of Biotechnology, Bhupat & Jyoti Mehta School of Biosciences, Indian Institute of Technology Madras, Chennai-600 036, India.

**Supplementary Table S1.** Endophytes isolated from the plant parts of Nothapodytes nimmoninana with their biomass and yield of camptothecin obtained.

| **S.No** | **Explant** | **Explant** | **Dried biomass obtained from suspension cultures (g/l)** | **Camptothecin yield (µg/g)** |
| --- | --- | --- | --- | --- |
| 1 | P1N-12-PE4 | Petiole | 8.6 ± 0.22 | 3.69 ± 0.3 |
| 2 | P1N-11-PE1 | Petiole | 10.0 ± 0.25 | 11.23 ± 2 |
| 3 | P1-2-LE3 | Leaf | 7.8 ± 0.20 | 3.69 ± 0.3 |
| 4 | P1N-12-LE2 | Leaf | 4.8 ± 0.12 | 0.48 ± 0.05 |
| 5 | P2-1-LE3 | Leaf | 8.8 ± 0.22 | 2.21 ± 0.7 |
| 6 | P1N-3-LE2 | Leaf | 10.0 ± 0.25 | 4.30 ±1.1 |
| 7 | P1N-12-PE3 | Petiole | 8.6 ± 0.22 | 3.33 ± 0.8 |
| 8 | P1N-11-PE2 | Petiole | 9.6 ± 0.24 | 0.21 ± 0.02 |
| 9 | P1N-6-LE2 | Leaf | 10.0 ± 0.25 | 1.37 ± 0.2 |
| 10 | P1N-5-LE1 | Leaf | 9.4 ± 0.24 | 4.18 ± 0.9 |
| 11 | P1N-5-LE2 | Leaf | 4.2 ± 0.11 | 0.25 ± 0.03 |
| 12 | P1-13-PE4 | Petiole | 6.6 ± 0.17 | 0.37 ± 0.03 |
| 13 | P1-4-LE3 | Leaf | 9.2 ± 0.23 | 5.57 ± 0.9 |
| 14 | P1N-2-LE1 | Leaf | 7.0 ± 0.18 | ND |
| 15 | P1-13-PE3 | Petiole | 6.6 ± 0.17 | 0.20 ± 0.02 |
| 16 | P2-2-LE1 | Leaf | 4.6 ± 0.12 | 2.79 ± 0.3 |
| 17 | P1-1-LE1 | Leaf | 8.0 ± 0.20 | 8.99 ± 1.2 |
| 18 | P1-2-LE1 | Leaf | 8.4 ± 0.21 | 10.89 ± 1.5 |
| 19 | P1-13-PE2 | Petiole | 9.0 ± 0.23 | 0.44 ± 0.04 |
| 20 | P2-12-BE1 | Bark | 8.2 ± 0.21 | 3.88 ± 0.4 |
| 21 | P1N-7-LE1 | Leaf | 9.6 ± 0.24 | 4.51 ± 0.4 |
| 22 | P1N-3-LE1 | Leaf | 8.8 ± 0.22 | 1.91 ± 0.1 |
| 23 | P1N-4-LE2 | Leaf | 8.4 ± 0.21 | 2.75 ± 0.3 |
| 24 | P1-2-LE2 | Leaf | 10.0 ± 0.25 | 2.12 ± 0.2 |
| 25 | P1N-4-LE1 | Leaf | 8.2 ± 0.21 | 1.14 ± 0.1 |
| 26 | P1N-6-LE1 | Leaf | 6.4 ± 0.16 | 7.47 ± 0.8 |
| 27 | P1N-7-LE2 | Leaf | 8.2 ± 0.21 | ND |
| 28 | PIN-7-LE4 | Leaf | 6.0 ± 0.15 | 1.67 ± 0.1 |
| 29 | P1-3-LE1 | Leaf | 7.0 ± 0.18 | 2.46 ± 0.4 |
| 30 | P1N-12-LE1 | Leaf | 9.8 ± 0.25 | 3.62 ± 0.3 |
| 31 | P1-8-LE2 | Leaf | 8.6 ± 0.22 | 0.67 ± 0.1 |
| 32 | P1N-8-LE3 | Leaf | 10.4 ± 0.26 | 1.56 ± 0.2 |
| 33 | P1-7-LE3 | Leaf | 9.0 ± 0.23 | 4.40 ± 1.2 |
| 34 | P1-5-LE3 | Leaf | 9.6 ± 0.24 | 5.75 ± 0.9 |
| 35 | P2-3-LE2 | Leaf | 11.0 ± 0.28 | ND |
| 36 | P2-9-PE1 | Petiole | 8.6 ± 0.22 | 4.57 ± 0.9 |
| 37 | P1N-9-LE2 | Leaf | 10.0 ± 0.25 | 5.10 ± 1.1 |
| 38 | P2-9-PE2 | Petiole | 11.0 ± 0.28 | 3.91 ± 0.8 |
| 39 | P2-8-PE2 | Petiole | 11.0 ± 0.28 | 0.86 ± 0.1 |
| 40 | P1N-9-LE1 | Leaf | 4.6 ± 0.12 | ND |
| 41 | P2-5-LE2 | Leaf | 6.8 ± 0.17 | ND |
| 42 | P2-5-LE3 | Leaf | 6.2 ± 0.16 | 0.56 ± 0.1 |
| 43 | P1N-8-LE1 | Leaf | 6.2 ± 0.16 | ND |
| 44 | P1N-10-LE4 | Leaf | 8.8 ± 0.22 | ND |
| 45 | P2-6-LE4 | Leaf | 11.6 ± 0.29 | 1.45 ± 0.3 |
| 46 | P1N-11-PE4 | Petiole | 8.8 ± 0.22 | 0.28 ± 0.05 |
| 47 | P2-7-LE1 | Leaf | 7.2 ± 0.18 | 12.32 ± 2.6 |
| 48 | P2-7-LE3 | Leaf | 11.0 ± 0.28 | 2.71 ± 0.7 |
| 49 | P2-7-LE2 | Leaf | 10.2 ± 0.26 | 7.71 ± 1.2 |
| 50 | P2-6-LE2 | Leaf | 10.6 ± 0.27 | 2.39 ± 0.4 |
| 51 | P1N-10-LE1 | Leaf | 5.4 ± 0.14 | ND |
| 52 | P2-5-LE4 | Leaf | 6.6 ± 0.17 | 2.01 ± 0.3 |
| 53 | P2-5-LE1 | Leaf | 7.2 ± 0.18 | ND |
| 54 | P2-8-PE4 | Petiole | 6.4 ± 0.16 | 4.35 ± 0.8 |
| 55 | P2-8-PE3 | Petiole | 9.2 ± 0.23 | 1.73 ± 0.2 |
| 56 | P2-9-PE3 | Petiole | 8.2 ± 0.21 | ND |
| 57 | P1-5-LE1 | Leaf | 7.0 ± 0.18 | 1.08 ± 0.2 |
| 58 | P2-8-PE1 | Petiole | 5.2 ± 0.13 | 1.41 ± 0.3 |
| 59 | P1-5-LE2 | Leaf | 7.0 ± 0.18 | ND |
| 60 | P1N-1-LE1 | Leaf | 6.0 ± 0.15 | 0.16 ± 0.02 |
| 61 | P1N-1-LE2 | Leaf | 9.0 ± 0.23 | 0.08 ± 0.01 |
| 62 | P1N-1-LE3 | Leaf | 7.6 ± 0.29 | 0.20 ± 0.04 |
| 63 | P1N-1-LE4 | Leaf | 10.2 ± 0.26 | 0.09 ± 0.02 |
| 64 | P1N-1-LE6 | Leaf | 7.8 ± 0.20 | 1.16 ± 0.2 |
| 65 | P1N-10-LE2 | Leaf | 8.4 ± 0.11 | 0.42 ± 0.1 |
| 66 | P2-6-LE2 | Leaf | 8.2 ± 0.21 | 0.18 ± 0.03 |
| 67 | P1-13-PE1 | Petiole | 7.4 ± 0.19 | 19.98 ± 1.7 |
| 68 | P2-12-BE4 | Bark | 7.8 ± 0.20 | 3.35 ± 0.8 |
| 69 | P2-2-LE4 | Leaf | 7.0 ± 0.18 | 0.13 ± 0.03 |
| 70 | P2-11-BE2 | Bark | 10.2 ± 0.26 | ND |
| 71 | P1-3-LE4 | Leaf | 8.4 ± 0.21 | 0.13 ± 0.02 |
| 72 | P2-11-BE3 | Bark | 9.4 ± 0.24 | ND |
| 73 | P2-15-SE3 | Stem | 8.6 ± 0.42 | 0.45 ± 0.1 |
| 74 | P2-15-SE4 | Stem | 7.8 ± 0.20 | 0.23 ± 0.04 |
| 75 | P2-15-SE2 | Stem | 8.2 ± 0.21 | 5.41 ± 0.9 |
| 76 | P2-15-SE1 | Stem | 9.2 ± 0.23 | 0.29 ± 0.03 |
| 77 | P2-10-BE1 | Bark | 10.0 ± 0.25 | ND |
| 78 | P2-10-BE2 | Bark | 9.2 ± 0.23 | 0.30 ± 0.04 |
| 79 | P2-14-SE1 | Stem | 7.8 ± 0.20 | ND |
| 80 | P2-11-BE1 | Bark | 8.2 ± 0.21 | ND |
| 81 | P2-3-LE1 | Leaf | 7.6 ± 0.19 | ND |
| 82 | P2-12-BE2 | Bark | 9.4 ± 0.24 | ND |
| 83 | P2-12-BE5 | Bark | 8.4 ± 0.21 | ND |
| 84 | P2-13-BE3 | Bark | 7.8 ± 0.20 | ND |
| 85 | P2-2-LE2 | Leaf | 7.4 ± 0.19 | 0.57 ± 0.03 |
| 86 | P2-2-LE3 | Leaf | 9.6 ± 0.24 | ND |
| 87 | P1N-1-LE5 | Leaf | 8.6 ± 0.22 | 0.10 ± 0.03 |
| 88 | P1N-1-LE7 | Leaf | 10.4 ± 0.26 | 0.51 ± 0.1 |
| 89 | P1N-1-LE8 | Leaf | 9.4 ± 0.24 | 0.88 ± 0.2 |
| 90 | P5-2-LE1 | Leaf | 7.2 ± 0.18 | 2.28 ± 0.7 |
| 91 | P5-2-LE2 | Leaf | 9.8 ± 0.45 | 0.72 ± 0.1 |
| 92 | P6-3-LE1 | Leaf | 8.8 ± 0.22 | 3.21 ± 0.8 |
| 93 | P5-6-PE3 | Petiole | 7.8 ± 0.20 | ND |
| 94 | P5-5-LE3 | Leaf | 9.6 ± 0.24 | 0.96 ± 0.2 |
| 95 | P4-2-LE2 | Leaf | 7.0 ± 0.18 | 0.20 ± 0.03 |
| 96 | P4-2-LE3 | Leaf | 10.0 ± 0.25 | 6.45 ± 0.9 |
| 97 | P5-4-LE3 | Leaf | 9.0 ± 0.23 | 1.62 ± 0.3 |
| 98 | P5-5-LE2 | Leaf | 8.6 ± 0.22 | ND |
| 99 | P6-3-LE2 | Leaf | 9.4 ± 0.24 | ND |
| 100 | P5-4-LE1 | Leaf | 6.8 ± 0.17 | ND |
| 101 | P5-4-LE2 | Leaf | 10.2 ± 0.26 | ND |
| 102 | P6-5-BE1 | Bark | 9.2 ± 0.23 | 0.14 ± 0.03 |
| 103 | P6-5-BE2 | Bark | 6.8 ± 0.11 | 0.56 ± 0.1 |
| 104 | P6-5-BE3 | Bark | 9.0 ± 0.23 | 0.83 ± 0.1 |
| 105 | P4-4-LE1 | Leaf | 9.6 ± 0.24 | 52.31 ± 12.6 |
| 106 | P5-6-PE1 | Petiole | 7.8 ± 0.20 | ND |
| 107 | P6-2-SE1 | Stem | 8.8 ± 0.22 | ND |
| 108 | P6-2-SE2 | Stem | 7.0 ± 0.18 | 9.40 ± 0.8 |
| 109 | P6-2-SE3 | Stem | 9.2 ± 0.23 | 0.81 ± 0.1 |
| 110 | P4-4-LE2 | Leaf | 8.8 ± 0.12 | 405.50 ± 34.7 |
| 111 | P4-4-LE3 | Leaf | 10.2 ± 0.26 | 1.67 ± 0.2 |
| 112 | P4-6-PE3 | Petiole | 8.6 ± 0.22 | ND |
| 113 | P5-1-LE1 | Leaf | 7.2 ± 0.18 | 1.50 ± 0.3 |
| 114 | P5-4-LE1 | Leaf | 9.6 ± 0.24 | 73.73 ± 9.5 |
| 115 | P4-2-LE1 | Leaf | 9.0 ± 0.23 | 7.45 ± 0.9 |
| 116 | P4-3-LE2 | Leaf | 10.4 ± 0.26 | ND |
| 117 | P4-1-LE3 | Leaf | 8.4 ± 0.21 | 0.75 ± 0.1 |
| 118 | P5-3-LE3 | Leaf | 7.4 ± 0.19 | ND |
| 119 | P6-4-BE2 | Bark | 9.6 ± 0.24 | 0.67 ± 0.1 |
| 120 | P4-3-LE1 | Leaf | 9.2 ± 0.23 | ND |
| 121 | P4-1-LE1 | Leaf | 8.6 ± 0.52 | 253.67 ± 35.8 |
| 122 | P4-1-LE2 | Leaf | 8.2 ± 0.21 | ND |
| 123 | P4-3-LE3 | Leaf | 7.6 ± 0.19 | 1.11 ± 0.2 |
| 124 | P5-1-LE3 | Leaf | 9.8 ± 0.25 | ND |
| 125 | P4-6-PE2 | Petiole | 6.4 ± 0.14 | 445.05 ± 41.3 |
| 126 | P5-3-LE1 | Leaf | 10.4 ± 0.26 | ND |
| 127 | P5-3-LE2 | Leaf | 8.0 ± 0.20 | 0.83 ± 0.2 |
| 128 | P5-5-LE1 | Leaf | 7.8 ± 0.20 | ND |
| 129 | P6-1-SE2 | Stem | 10.0 ± 0.52 | 40.95 ± 3.5 |
| 130 | P4-5-LE2 | Leaf | 8.6 ± 0.22 | 1.90 ± 0.3 |
| 131 | P4-5-LE3 | Leaf | 10.2 ± 0.26 | ND |
| 132 | P6-4-BE3 | Bark | 8.2 ± 0.21 | ND |

a)
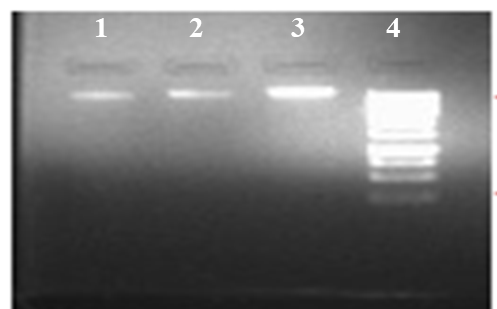
b)
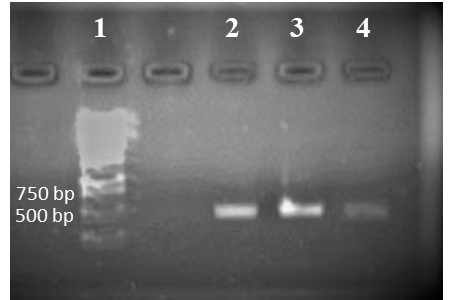


**Supplementary Figure** **S1.** Whole genomic DNA isolated from the endophytic strains (a) NCPT1 (Lane 1), NCPT2 (Lane 2), NCPT3 (Lane 3) and 1Kb DNA ladder (Lane 4). PCR amplification of the isolated DNA from non camptothecin producing endophytes using ITS1 and ITS4 primers (b) 1kb DNA ladder (Lane 1), Purified PCR products from NCPT1 (Lane 2), NCPT2 (Lane 3) and NCPT3 (Lane 4).


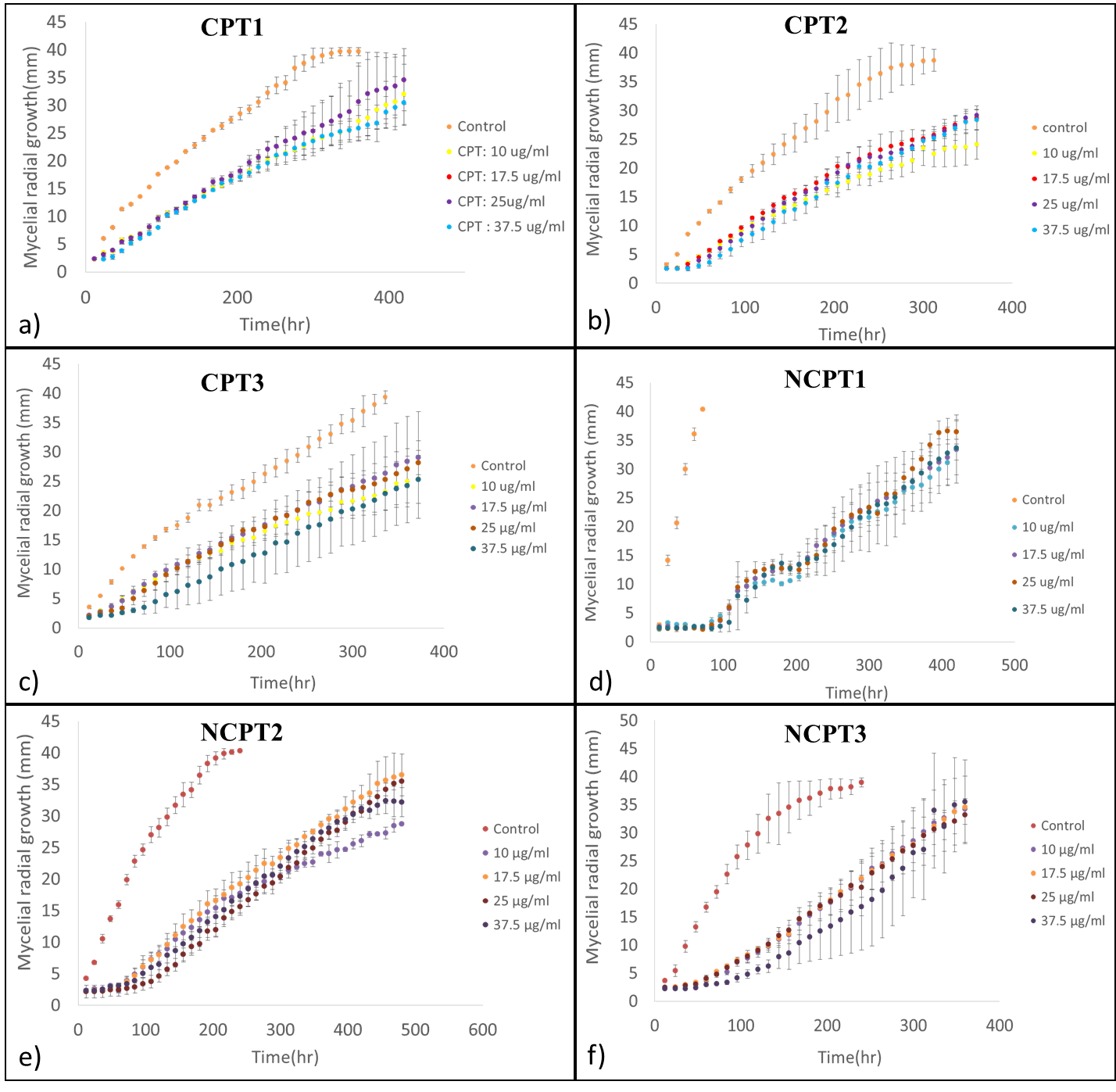


**Supplementary Figure** **S2.** Growth profile of camptothecin producing strains CPT1 (a), CPT2 (b) and CPT3 (c) and non camptothecin producing strains NCPT1 (d), NCPT2 (e) and NCPT3 (f) with increasing concentrations of standard camptothecin in media.

**
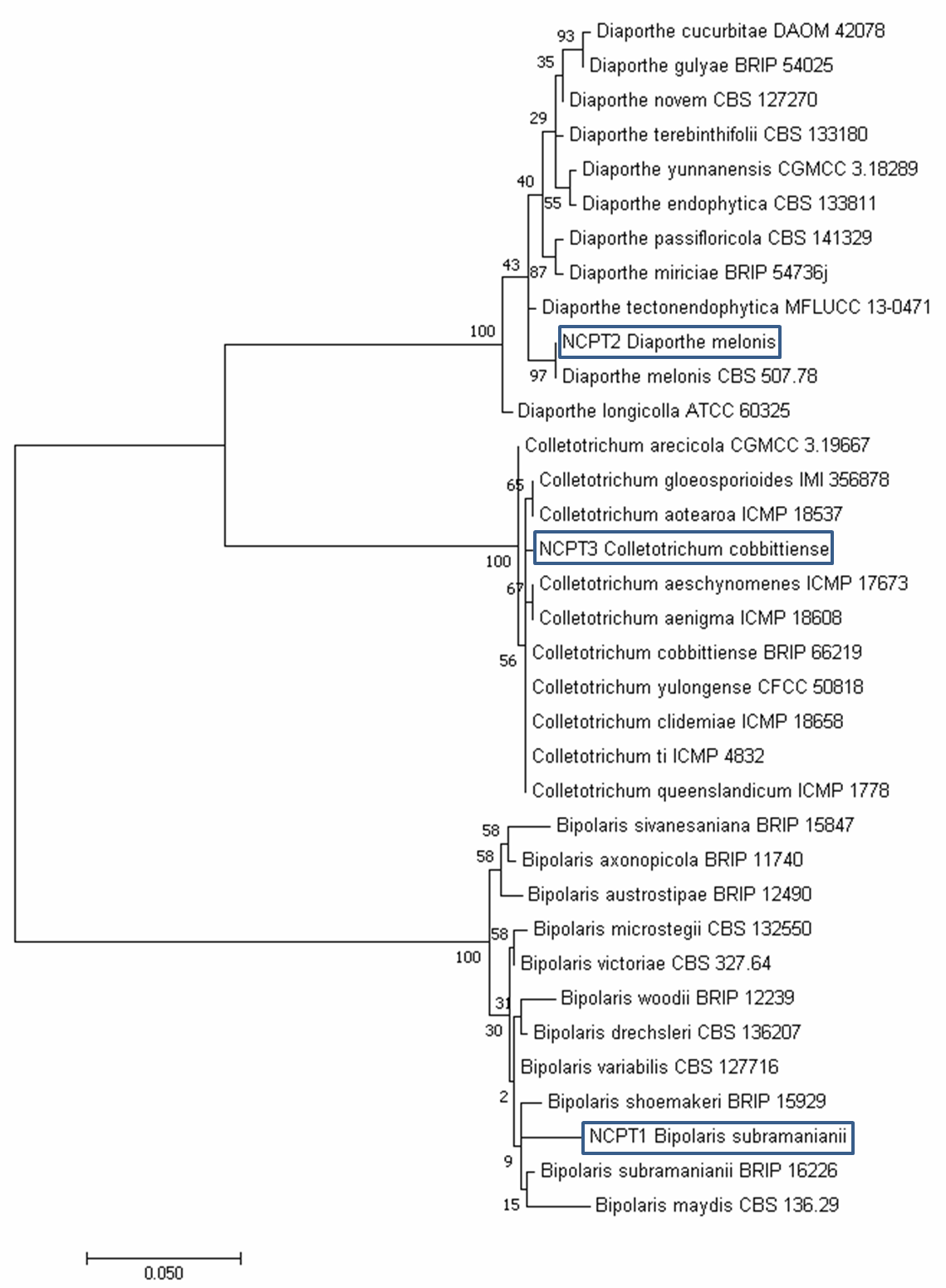
**

**Supplementary Figure** **S3.** Molecular Phylogenetic analysis by Maximum Likelihood method based on the Jukes-Cantor model. The strains isolated in this study are shown inside a blue rectangular box. The tree with the highest log likelihood (-1829.20) is shown. The percentage of trees in which the associated taxa clustered together is shown next to the branches. Initial tree(s) for the heuristic search were obtained automatically by applying Neighbor-Join and BioNJ algorithms to a matrix of pairwise distances estimated using the Maximum Composite Likelihood (MCL) approach, and then selecting the topology with superior log likelihood value. The tree is drawn to scale, with branch lengths measured in the number of substitutions per site. The analysis involved 35 nucleotide sequences. Codon positions included were 1st+2nd+3rd+Noncoding. All positions containing gaps and missing data were eliminated. There were a total of 450 positions in the final dataset. Evolutionary analyses were conducted in MEGA7.
